# Relationship between obstructive sleep apnoea syndrome and gastrointestinal diseases: a systematic review and Meta-analysis

**DOI:** 10.1038/s41533-024-00373-y

**Published:** 2024-05-25

**Authors:** Liubin Cao, Chengpei Zhou, Rupei Zhang, Shan Zhou, Xiaolei Sun, Jun Yan

**Affiliations:** 1 Department of Forensic Medicine, Nantong University Medical College, Nantong, China; 2 Department of Pathogenic Biology, Nantong University Medical College, Nantong, China

**Keywords:** Outcomes research, Respiratory distress syndrome

## Abstract

Studies exploring the association between obstructive sleep apnoea syndrome (OSA) and gastrointestinal diseases (GID) are important for enhancing clinical outcomes. This study aimed to systematically assess the association between these two diseases. Adhering to PRISMA guidelines, a comprehensive literature search was conducted across databases including PubMed, Web of Science, Willey Library, Cochrane Library and Scopus. This search focused on English literature published up to January 2024. Literature screening, quality assessment (using the NOS scale) and data extraction were performed by two independent researchers. Statistical analyses were performed using the meta-package of the R.4.2.2 software. An initial screening of 2178 papers was conducted and 11 studies were included. Meta-analysis results showed a significant association between OSA and GID (*p* < 0.01). Subgroup analyses further indicated a stronger association between OSA and GID in Asian populations compared to Europe and the United States. In addition, both benign and malignant GID were significantly associated with OSA, with a pronounced association for malignant GID than for benign GID. The results of publication bias analysis revealed no significant bias (Begg’s test *p* = 0.45, Egger’s test *p* = 0.60). This study uncovers a notable association between OSA and GID, especially in Asian populations, suggesting that clinicians should consider the potential connection between these two diseases during diagnosis and treatment. However, due to the heterogeneity and limitations of the study, these conclusions need to be further validated through more comprehensive research.

## Introduction

Obstructive sleep apnoea syndrome (OSA) is a prevalent sleep breathing disorder characterised by recurrent partial or complete obstruction of the upper airway during sleep, leading to apnoea or hypopnoea^1,2^. OSA not only diminishes sleep quality, but is also associated with a wide range of health problems such as cardiovascular disease, metabolic disorders, and neurocognitive dysfunction^3–5^, thereby emerging as an important public health concern.

Recently, the potential link between OSA and gastrointestinal diseases (GID) has garnered increasing attention from the medical community^6–10^. GID, including gastroesophageal reflux disease (GERD) and inflammatory bowel disease (IBD), commonly affects the health of the global population. It has been suggested that symptoms of GERD are more prevalent in patients with OSA, and these gastrointestinal symptoms may in turn exacerbate the manifestations of OSA. GID affects not only the digestive system but may also cause a range of systemic health problems.

In their Meta-analysis, Nabil El Hage Chehade and other researchers^7^ suggested an association between OSA and GERD, independent of screening or diagnostic methods. They noted that GERD’s presence does not directly affect the severity of OSA. This finding implies a more complex interaction mechanism between OSA and GID, possibly involving a variety of physiological and pathological processes including intermittent hypoxaemia^10,11^, nocturnal respiratory abnormalities^8^, and comorbid obesity and metabolic syndrome^12^.

Despite studies that have been conducted to explore the potential association between OSA and GID, the results of these studies have been inconsistent^6,13–22^. Therefore, this study aims to comprehensively evaluate the association between OSA and various types of GID to provide stronger evidence support for clinical diagnosis and treatment, thereby improving the diagnostic and treatment protocols and quality of life for patients.

## Results

### Results of literature screening

**Fig. 1:**
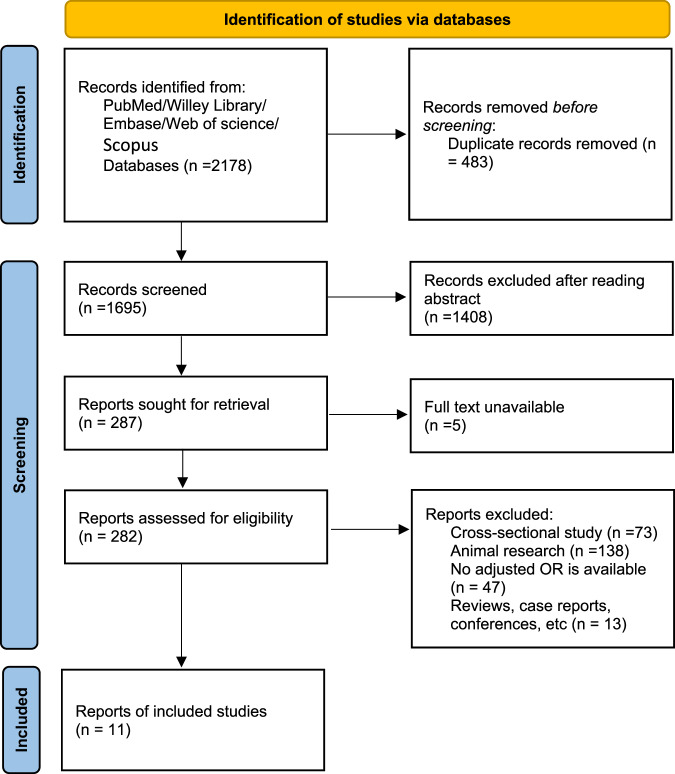
PRISMA diagram. Schematic diagram of the literature screening process.

The literature screening process for this study was meticulously executed in strict adherence to the pre-established inclusion and exclusion criteria. The initial search yielded a total of 2,178 pieces of literature, with 2,173 obtained through electronic database searches, and 5 obtained through manual searches from references. After removing 483 duplicates, 1408 pieces of literature were excluded due to titles and abstracts not aligning with the study’s focus and 5 pieces of literature were not accessible in full text. Subsequently, the remaining 282 literatures were then reviewed in full text, leading to the exclusion of an additional 271 papers, primarily due to non-compliance with the study design, or incomplete or substandard data quality. Ultimately, a total of 11 pieces of literature were eventually included in the final analysis, as seen in Fig. 1. Details of the included literature are given in Table 1.

**Table 1:** General information on the included literature.

Author	Year	Study design	Country	Sample size		Age, years		Male (%)		Alcohol		GID type	Malignant/benign
				GID	Non-GID	GID	Non-GID	GID	Non-GID	GID	Non-GID		
Chen^13^	2020	Cohort study	China	4180	16720	>20	>20	80	80	NR	NR	Colorectal cancer	Malignant
Cummings^14^	2013	Case–control study	USA	54	171	63 (11.1)	57 (13.6)	70.4	43.9	NR	NR	Barrett’s esophagus	Benign
Fang^15^	2015	Case-Control Study	China	14	630	NR	NR	NR	NR	NR	NR	Colon Cancer	Malignant
Ghiasi^16^	2017	Case-Control Study	Iran	100	97	51.97 (10.15)	38.15 (9.6)	65	45.3	NR	NR	Irritable bowel syndrome	Benign
Gozal^17^	2016	Case-Control Study	USA	3810	5242	NR	NR	NR	NR	NR	NR	Colon Cancer	Malignant
Hoffman^6^	2022	Case-Control Study	USA	114890	175770	>18	>18	42.4	44.5	NR	NR	Inflammatory bowel disease	Benign
Huang^18^	2021	Case-Control Study	USA	NR	NR	NR	NR	NR	NR	NR	NR	Colon/Rectum Cancer	Malignant
Kang^19^	2021	Case-Control Study	Korea	41	277	52.1 (11.2)	50.7 (12.8)	73.2	74.7	53	62.5	GRED	Benign
Valentin^20^	2023	Cohort study	France	102	102	NR	NR	NR	NR	NR	NR	Irritable bowel syndrome	Benign
Vela^21^	2014	Case-Control Study	USA	83	75	59.2 (9.4)	61.7 (6.7)	89	97	NR	NR	Barrett’s esophagus/GRED	Benign
You^22^	2014	Case-Control Study	Korea	231	776	55.8 (8.4)	54.9 (8.8)	63.5	38.5	64.3	69.1	GRED	Benign

Note: Gastrointestinal diseases (GID); Not reported (NR).

### Literature quality evaluation results

The quality of the literature included in this study was evaluated using the NOS scale. The evaluation revealed that seven literatures scored more than 7, which showed high research quality, and four literatures scored 6, which was medium quality and met the quality requirements of this paper, as shown in Table 2.

**Table 2:** Literature quality assessment (NOS scale).

Author	Year of publication	Case selection	Comparability	Conclusion	NOS score
Chen^13^	2020	4	2	2	8
Cummings^14^	2013	3	1	2	6
Fang^15^	2015	3	2	2	7
Ghiasi^16^	2017	4	2	2	8
Gozal^17^	2016	4	2	1	7
Hoffman^6^	2022	3	2	1	6
Huang^18^	2021	4	1	2	7
Kang^19^	2021	3	2	2	7
Valentin^20^	2023	4	1	1	6
Vela^21^	2014	3	2	2	7
You^22^	2014	3	2	1	6

### Association between OSA and GID

**Fig. 2:**
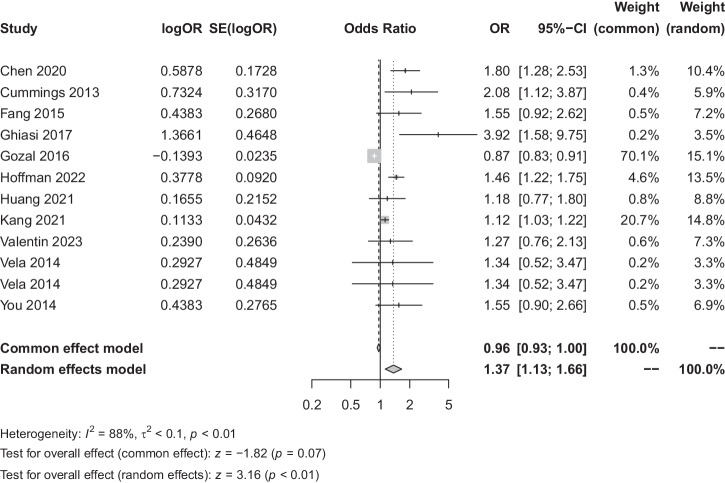
Forest plot of the relationship between OSA and GID. OR odds ratio, SE standard error, CI confidence interval, OSA obstructive sleep apnoea syndrome, GID gastrointestinal diseases.

**Fig. 3:**
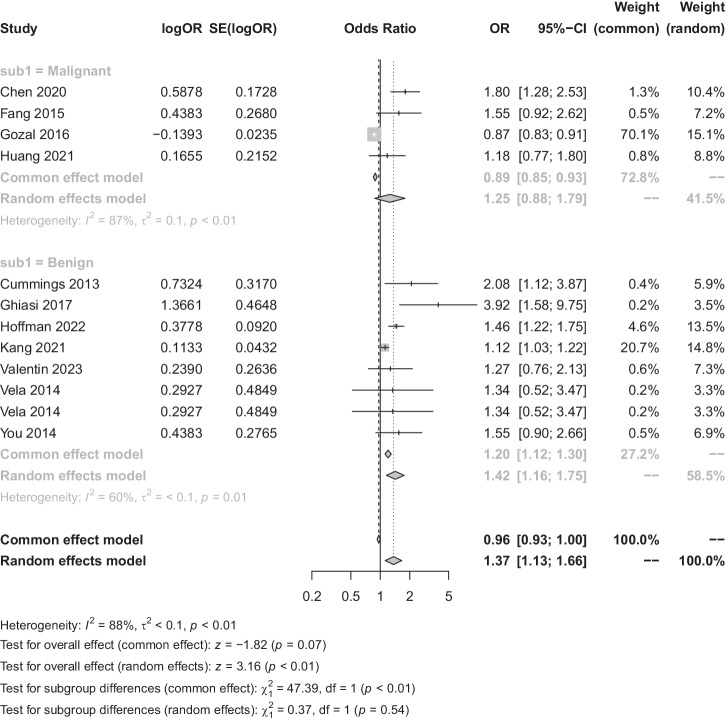
Forest plot of the relationship between OSA and GID (subgroup analysis of disease benignity and malignancy). OR odds ratio, SE standard error, CI confidence interval, OSA obstructive sleep apnoea syndrome, GID gastrointestinal diseases.

**Fig. 4:**
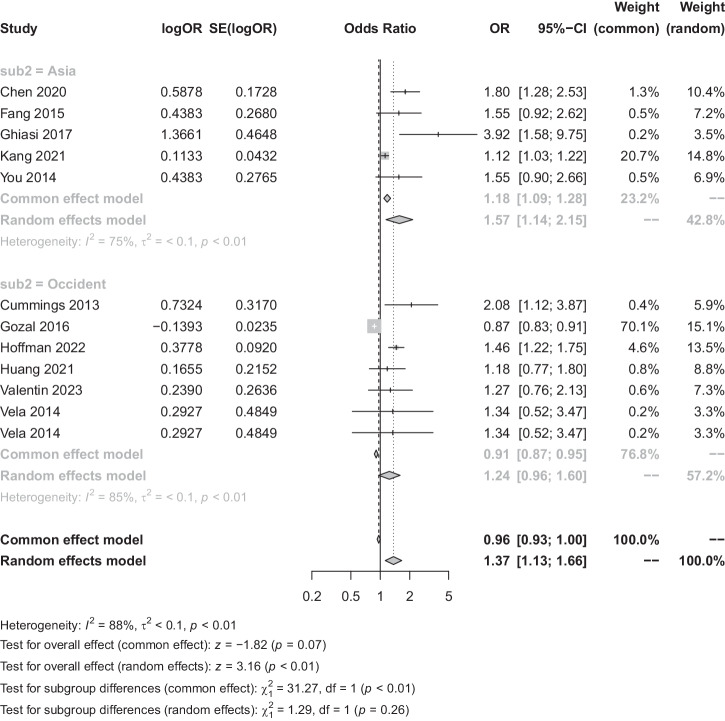
Forest plot of the relationship between OSA and GID (subgroup analysis of areas). OR odds ratio, SE standard error, CI confidence interval, OSA obstructive sleep apnoea syndrome, GID gastrointestinal diseases.

The meta-analysis incorporated eleven studies with a total of 2729 patients to assess the association between OSA and GID. Heterogeneity analysis found substantial variability *I*^*2*^ = 88%. By using a random-effects model, the Meta-analysis showed a statistically significant correlation between the two diseases (OR = 1.37, 95% CI [1.13, 1.66], *p* < 0.01) (Fig. 2). For subgroup analysis, GID was categorized into benign and malignant conditions, observing a significant difference (*p* < 0.01) between benign (OR = 1.25, 95% CI [0.88, 1.79]) and malignant GID (OR = 1.42, 95% CI [1.16, 1.75]). When analysed using a fixed-effects model, a notably stronger association was observed in malignant GID (Fig. 3). Subgroup analyses based on regional classification revealed that the correlation between OSA and GID was significantly different between geographic regions using fixed-effect model analysis (*P* < 0.01) (Fig. 4), with the correlation between OSA and GID being stronger in Asia (OR = 1.57, 95% CI [1.14, 2.15]) than Europe and the United States (OR = 1.24, 95% CI [1.13, 1.66]) was stronger (Fig. 4).

### Factors influencing the correlation between OSA and GID

**Fig. 5:**
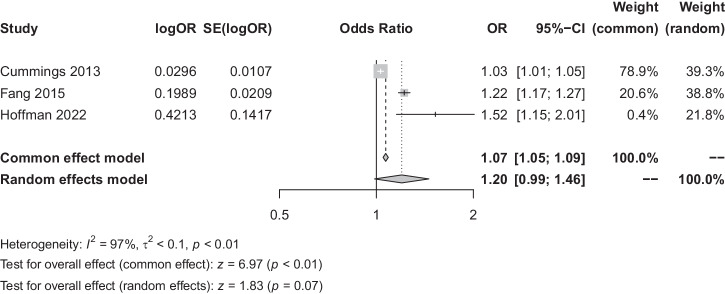
Factors influencing the correlation between OSA and GID (age). OR odds ratio, SE standard error, CI confidence interval, OSA obstructive sleep apnoea syndrome, GID gastrointestinal diseases.

**Fig. 6:**
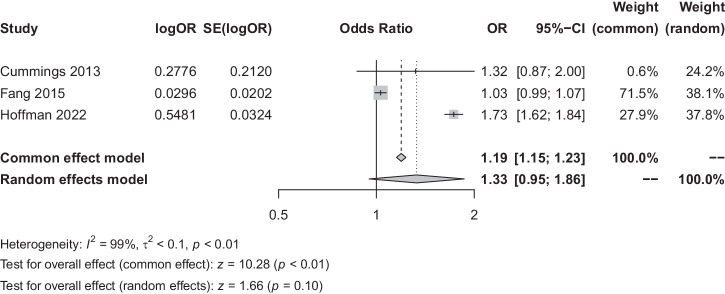
Factors influencing the correlation between OSA and GID (gender). OR odds ratio, SE standard error, CI confidence interval, OSA obstructive sleep apnoea syndrome, GID gastrointestinal diseases.

In analysing the factors influencing the OSA-GID correlation, both age and gender demonstrated a high degree of heterogeneity for both the age and gender factors (*I*^*2*^ = 97% and *I*^*2*^ = 99%), both analysed using a random-effects model. The findings suggested that neither age (OR = 1.20, 95% CI [0.99, 1.46], *P* = 0.07) nor gender (OR = 1.33, 95% CI [0.95, 1.86], *P* = 0.10) were not key factors influencing the correlation between OSA and GID, as seen in Figs. 5, 6.

### Publication bias analysis

**Fig. 7:**
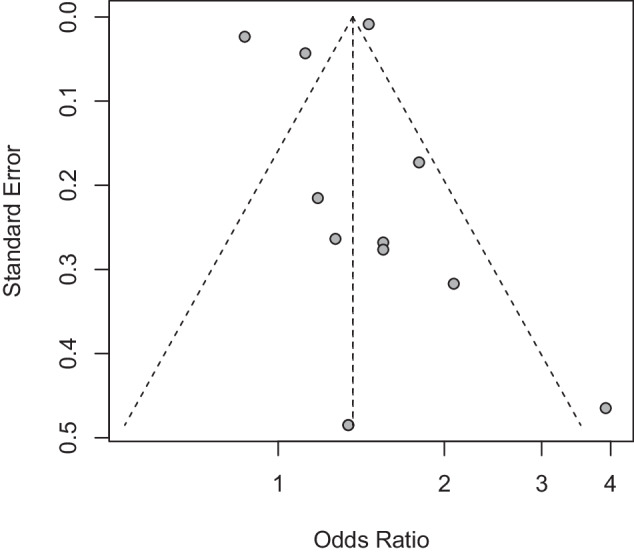
Funnel plot of the relationship between OSA and GID. OSA obstructive sleep apnoea syndrome, GID gastrointestinal diseases.

To assess the possible presence of publication bias in this Meta-analysis, several statistical methods were employed. Visual inspection of funnel plots indicated good symmetry, despite some studies falling outside the funnel. Additionally, the outcomes of Begg’s test (*z* = 0.76, *p* = 0.45) and Egger’s test (*t* = −0.54, *p* = 0.60) showed that the included literature was free of publication bias, as seen in Fig. 7.

### Sensitivity analysis

The results of the sensitivity analyses showed that neither the combined effect sizes nor the heterogeneity indicators of the Meta-analyses changed significantly after the exclusion of any single study, suggesting that the results of our Meta-analyses were highly robust and that no single study had an excessive effect on the overall results.

## Discussion

The results of this systematic evaluation and Meta-analysis suggest that there is a strong interconnection between OSA and GID, especially for malignant GID. This finding provides new insights into the mechanisms of OSA and GID interactions and suggests the need for optimisation in clinical practice.

Numerous studies have been conducted in recent years to investigate the mechanisms linking OSA and GID and to establish a causal relationship between^2,3,23^. Recent studies have revealed that OSA-induced physiological changes, such as intermittent hypoxaemia and sleep disruption, may increase the risk of GID, including gastroesophageal reflux disease, gastric ulcers, and another digestive disorders^20–22^. The increased stress on the body’s internal environment caused by OSA may precipitate gastrointestinal dysfunction^18,19^. Furthermore, the high prevalence of obesity and metabolic syndrome among OSA patients may also further exacerbate the risk of GID^24^. However, some studies have yielded inconsistent results^6,17–19^. Therefore, our systematic evaluation and meta-analysis aimed to provide more comprehensive and precise evidence by synthesising data from a large number of studies to address the existing controversies regarding the association of OSA with GID. We focused on parsing differences across regions, ethnicities, and lifestyle contexts, and on exploring potential biological mechanisms, aiming to offer deeper insights into the field. Our study not only focuses on the aggregation of existing evidence but also endeavours to identify potential biases in research and suggest future research directions to provide more instructive information for clinical practice and patient management.

From a pathophysiological perspective, the correlation between OSA and GID may involve a series of complex biological pathways. Intermittent hypoxaemia, which often occurs at night in patients with OSA, may trigger a systemic inflammatory response. This inflammatory response is particularly closely related to benign GID, which implies that OSA may cause damage to the gastrointestinal mucosal barrier and increase the probability of GERD and other benign GID^4,25^. In addition, OSA-induced changes in intrathoracic and intra-abdominal pressure may disrupt the lower oesophageal sphincter’s function, promoting gastric acid reflux^26^. These changes can directly impair gastrointestinal health and may also indirectly affect the digestive system through neuroreflex mechanisms. The prevalence of obesity in patients with OSA is also an important factor exacerbating the risk of GID. Obesity may exacerbate the symptoms of GID by increasing the intra-abdominal pressure and causing metabolic disturbances^27^. In addition, obesity may also affect the rate of gastric emptying and the balance of intestinal flora, which may further exacerbate gastrointestinal problems^28^.

In exploring the geographic distribution of the OSA-GID relationship, our study found a particularly significant association in Asian populations^13,15,19^. This pattern may be due to the unique genetic background, dietary habits, and lifestyle of the Asian region. The high content of salt and fat in the Asian diet may significantly influence the pathogenesis of GID, such as GERD^18^, promoting increased gastric acid secretion and exacerbating GERD symptoms, which may trigger or worsen GID in patients with OSA. In addition, the high carbohydrate intake in Asian populations may be associated with an increased prevalence of GID, especially among patients with diabetes mellitus^29^. Genetic factors There may be specific genetic variants or genetic predispositions in the development of OSA and GID that make Asian populations more susceptible to both diseases. For example, genetic variants associated with obesity, diabetes, and other metabolic diseases may increase the risk of both OSA and GID^30^. Nevertheless, research in this domain is nascent, and more comprehensive genetic epidemiological studies are needed to uncover specific molecular mechanisms and genetic predispositions.

These findings highlight the importance of identifying and managing patients with both OSA and GID in daily clinical practice. Primary care physicians should consider the interrelationship between OSA and GID, particularly in Asian, and conduct a comprehensive evaluation of the medical history including pay attention to the patient’s dietary habits, lifestyle, and genetic background. It is recommended that during the diagnosis, a comprehensive evaluation should be performed for GID patients with OSA symptoms. Multi-disciplinary treatment should be considered for these patients, including Department of Gastroenterology and Department of Respiration.

This study analyzed the association between OSA and GID, and explored the association between OSA and malignant or benign GID through subgroup analysis, as well as subgroup analysis of the population, to obtain more comprehensive analysis results. Although there are some limitations. Most included studies were observational, causality could not be established. In addition, the high heterogeneity of the included studies may affect the robustness and generalisability of the results, and future studies should adopt a more rigorous study design and uniform diagnostic criteria to further validate our findings.

In conclusion, this study not only highlights the importance of considering patients’ gastrointestinal conditions in the diagnosis and treatment of OSA, but also sheds light on biological mechanisms that need to be investigated in greater depth. Future studies should aim to clarify the causal relationship between OSA and GID, analyse regional and racial differences, and delve deeper into the underlying molecular and cellular mechanisms, so as to provide more comprehensive treatment strategies for the clinic.

## Methods

As this study involves the summary and analysis of other studies, it does not involve medical ethics approval or patient-informed consent.

### Search strategy

This study was conducted in accordance with the PRISMA guidelines^23^, and to systematically assess the relationship between OSA and GID, the study was searched in several databases, including PubMed, Web of science, Willey Library, Cochrane Library, and Scopus. The search strategy included keywords and medical subject terms related to OSA and various GID. The search language was English, and the search period was from the establishment of each database to January 2024.

The search terms included “Obstructive Sleep Apnea”, “OSA”, “Gastrointestinal Diseases”, “Gastroesophageal Reflux Disease (GERD)”, “Irritable Bowel Syndrome (IBS)”. “Inflammatory Bowel Disease (IBD)”, “Crohn’s Disease”, “Ulcerative Colitis “, “Gastritis”, “Peptic Ulcer Disease”, “Gastroenteritis “, “Colorectal Cancer”. The search was performed using a combination of subject terms and free words with matching truncation, using PubMed as an example: (“Obstructive Sleep Apnea” [MeSH Terms] OR “OSA” [Title/Abstract]) AND (“Gastrointestinal Diseases” [MeSH Terms] OR “Gastroesophageal Reflux Disease (GERD)”[Title/Abstract] OR “Irritable Bowel Syndrome (IBS)”[Title/Abstract] OR “Inflammatory Bowel Disease (IBD)”[Title/Abstract] OR “Crohn’s Disease”[Title/Abstract] OR “Ulcerative Colitis”[Title/Abstract] OR “Gastritis”[Title/Abstract] OR “Peptic Ulcer Disease”[Title/Abstract] OR “Gastroenteritis”[Title/Abstract] OR “Colorectal Cancer”[Title/Abstract]).

### Literature inclusion and exclusion criteria

Inclusion criteria included (1) studies with a clear diagnosis of OSA, and the basis for the diagnosis should include standard sleep monitoring data or recognised clinical diagnostic guidelines; (2) studies assessed the association between OSA and GID (such as GERD, IBS, IBD, Crohn’s Disease, Ulcerative Colitis); (3) studies provided quantitative data, such as risk ratios (RR), odds ratios (OR), or correlation data; (4) the publication was a peer-reviewed full-text article; and (5) the article was written in English.

Exclusion criteria included: (1) interventional studies, case reports, commentaries, expert opinions, conference abstracts, review articles, or non-peer-reviewed publications; (2) studies that did not provide sufficient data to estimate risk ratios or odds ratios; (3) studies in which the diagnostic criteria for OSA or GID were unclear or did not meet widely accepted clinical guidelines; (4) studies that were repetitively published or had duplicated data; (5) studies that were not original, such as secondary data based on published data.

### Literature screening

The literature screening process for this study followed a rigorous process, wherein the titles and abstracts of retrieved literature were initially examined by two intended researchers. The preliminary screening aimed to exclude irrelevant studies, specifically those not addressing OSA or a specific GID. Subsequently, a detailed full-text review was undertaken for literature that seems to the inclusion criteria, to determine whether the inclusion criteria were fully met. Discrepancies in opinion were resolved through discussion or, if necessary, by consulting third-party experts.

### Literature quality assessment

The Newcastle-Ottawa Scale (NOS), a standardised tool for appraising the quality of observational studies, particularly cohort and case-control studies, was used in this study for literature quality evaluation. The NOS encompasses three main domains: selectivity (choice of the study population), comparability (comparisons between study groups), and outcome (assessment of the study’s results.) The NOS scale reflects the methodological quality of each study by providing it with an overall score of up to 9 points. In this study, each study meeting the inclusion criteria was independently assessed by two researchers using the NOS scale. Disagreements during the scoring process were addressed through discussion, and third-party experts were consulted as needed.

### Data extraction

In this study, the data extraction process was carried out independently by two researchers to ensure the accuracy and completeness of the data. A comprehensive data extraction form was designed to gather key information from each study, including (1) basic information about the study, such as authors, year of publication, and type of study design; (2) characteristics of the study population, including sample size, age range, and gender ratio; (3) primary and secondary outcome indicators, including all relevant clinical outcomes and measurements; (4) study results, such as the odds ratio (OR), correlation coefficient, and so on, as well as their confidence intervals and statistical significance levels. Upon completion of each data extraction, investigators cross-verified the data to eliminate potential errors or biases. Any disagreements identified during the data extraction process were resolved through discussion, and third-party expert consultation was sought as necessary.

### Statistical methods

The Meta-analysis was conducted using the meta-package in R software (R 4.2.2). Firstly, we will extract the corrected Odds Ratio (OR) of the outcome metrics and their 95% Confidence Interval (CI) from each included study. Subsequently, these ORs and 95% CIs were converted to logORs and their standard errors (Standard Error, SE) for meta-analysis. For the heterogeneity test, *I*² statistic was used to assess the heterogeneity between studies, when *I*² ≤ 50%, indicating small heterogeneity, a fixed-effect model (FEM) was employed for meta-analysis. If *I*² > 50%, indicating a high degree of heterogeneity, in which case a random-effects model was used to analyse the data. To assess the quality of the studies, the Newcastle-Ottawa Scale (NOS) was used for cohort studies. In addition, for studies numbering 10 or more, funnel plots, Begg’s test, and Egger’s test were utilized to assess possible publication bias. A statistically significant difference of *P* < 0.05 was used as the criterion for all tests.

## Data Availability

The data used to support the findings of this study are included within the article.
